# Association of variation in the *LAMA3* gene, encoding the alpha-chain of laminin 5, with atopic dermatitis in a German case–control cohort

**DOI:** 10.1186/1471-5945-14-17

**Published:** 2014-11-03

**Authors:** Susanne Stemmler, Qumar Parwez, Elisabeth Petrasch-Parwez, Joerg T Epplen, Sabine Hoffjan

**Affiliations:** 1 Department of Human Genetics, Ruhr-University, Universitätsstrasse 150, 44801 Bochum, Germany; 2 Private medical practice, Gladbeck, Germany; 3 Department of Neuroanatomy and Molecular Brain Research, Ruhr-University Bochum, Bochum, Germany; 4 Faculty of Health, Witten/Herdecke University, Witten, Germany

**Keywords:** Atopic dermatitis, Genetic factors, Laminin 5, *LAMA3*, Skin barrier function

## Abstract

**Background:**

Atopic dermatitis (AD) is a chronic inflammatory skin disorder caused by complex interaction of genetic and environmental factors. Besides mutations in the filaggrin gene, leading to impaired skin barrier function, variation in genes encoding additional skin proteins has been suggested to contribute to disease risk. Laminin 5, playing an important role in skin integrity, is composed of three subunits encoded by the *LAMA3*, *LAMB3* and *LAMC2* genes in which biallelic mutations cause epidermolysis bullosa junctionalis. We aimed at evaluating the role of variation in the *LAMA3*, *LAMB3* and *LAMC2* genes for AD pathogenesis.

**Methods:**

29 single nucleotide polymorphisms (SNPs) were genotyped in the three genes in a German AD case–control cohort comprising 470 unrelated AD patients and 320 non-atopic controls by means of restriction enzyme digestion. Allele, genotype and haplotype frequencies were compared between cases and controls using chi-square testing and the Haploview software.

**Results:**

Several SNPs in the *LAMA3* gene showed significant association with AD in our cohort (p <0.01), while we did not detect association with variations in the *LAMB3* and *LAMC2* genes. Haplotype analysis additionally revealed several significantly associated haplotypes in the *LAMA3* gene. Due to extensive linkage disequilibrium, though, we were not able to further differentiate the specific disease causing variation(s) in this region.

**Conclusions:**

We established the *LAMA3* gene as novel potential susceptibility gene for AD. Additional studies in independent cohorts are needed to replicate these results.

## Background

Atopic dermatitis (AD) is a chronic inflammatory skin disorder caused by complex interaction between genetic and environmental factors [1]. Besides dysregulated immune mechanisms that have long been suspected to play a major role for AD pathogenesis, the impact of an intact skin barrier in the protection against AD has gained increasing attention over the recent years [2]. In particular, the role of filaggrin, a major structural protein in the stratum corneum of the epidermis, has been extensively studied. Mutations in the *FLG* gene, located in the epidermal differentiation complex (EDC) on chromosome 1q21 [3], have consistently been associated with early-onset persistent AD [4]. It could be demonstrated that *FLG* mutations constitute the most significant known risk factor for AD development so far [5]. However, impaired skin barrier function has also been shown in AD patients without *FLG* mutations [6], suggesting that variation in genes encoding additional skin proteins may play a role in AD pathogenesis.

Laminin 5 is another protein that plays an important role for skin integrity [7]. It is comprised of three different subunits, built by the LAMA3 (alpha-3), LAMB3 (beta-3) and LAMC2 (gamma-2) polypeptide chains. Laminin 5 (also called laminin-332) is involved in connecting dermis and epidermis and induces adhesion, spreading and migration of human keratinocytes [8]. The three polypeptide chains are encoded by the *LAMA3*, *LAMB3* and *LAMC2* genes on chromosomes 18q11.2, 1q32.2 and 1q25.3, resp. [9]. Biallelic mutations in each of these three genes are known to cause epidermolysis bullosa junctionalis, a severe (type Herlitz) or less severe (type Non-Herlitz) skin disorder characterized by blisters and erosions of the skin [10]. Furthermore, laminin 5 synthesis is elevated during acute wound healing in healthy individuals [11].

Given the important role of mutations in skin barrier proteins in AD pathogenesis, we hypothesized that variation in one or more of the laminin 5 subunits may also confer risk for AD. Therefore we evaluated 29 single nucleotide polymorphisms (SNPs) in the three genes in a German AD case–control cohort and present first evidence that the *LAMA3* gene may be a novel susceptibility gene for AD.

## Methods

### Subjects

470 unrelated patients with AD with a mean age of 19 ± 15 years (median 11 years) were recruited by a consultant specialist for AD (Q.P., Gladbeck, Germany) as described before [12]. AD diagnosis was established based on the criteria by Hanifin and Rajka [13]. Since the risk remains very high for primarily asymptomatic children to develop an allergic disease during childhood or even adulthood [14,15], we chose to use non-allergic adults as a control group. Therefore, 320 individuals of at least 40 years (mean age 62 ± 11 years, median 63 years) that had neither self-reported allergies or allergic symptoms nor first degree relatives with allergic diseases were recruited in the same private practice as the patients. The controls further underwent clinical examination in order to exclude symptoms of AD, asthma or allergic rhinitis (see [12]). All participants were Germans of European ancestry and gave informed consent prior to enrolment. The Declaration of Helsinki protocols were followed and the study was approved by the Ethics Committee of the Ruhr-University Bochum.

### Genotyping

DNA of AD patients and controls was extracted as described before [16]. We selected 29 SNPs in the three genes (17 in the *LAMA3* gene, 8 in *LAMB3,* and 4 in *LAMC2)* that represented the haplotype block structures according to HapMap [17]. Genotyping was performed by polymerase chain reaction (PCR) followed by restriction enzyme digestion. PCR was done in a total volume of 10 μl, containing 40 ng DNA, 200 mmol of each dNTP, 1.5-3 mmol MgCl_2_, 5 pmol of each primer, and 0.5 U Taq-DNA-polymerase (Genecraft, Münster, Germany) on the RoboCycler or Biometra *T* cycler (Stratagene, Heidelberg, Germany and Biometra GmbH, Göttingen, Germany, respectively). After two initial cycles at 6° C and 3°C above the annealing temperature, 28–32 cycles of 95°C (30 sec), annealing temperature (30 sec) and 72°C (30 sec) were run. PCR products were subsequently digested with the respective restriction enzyme, the fragments separated on 2.5%-3.5% agarose gels in 1xTBE buffer (30–60 min, 200 V) and visualized with ethidium bromide (0.5% [w/v]). Additional information about primer sequences, PCR conditions and restriction enzymes is summarized in Additional file 1.

### Statistics

Genotype and allele frequencies were compared between AD patients and controls according to the χ2 method; the significance threshold was set at p < 0.05. We evaluated every SNP for deviations from Hardy-Weinberg equilibrium (HWE) using the deFinetti program [18]. The program Haploview 4.0 [19] was used to estimate haplotype frequencies and test for haplotypic association. We applied Bonferroni correction for multiple tests; however, since this approach has been controversially discussed for genetic case–control studies [20], especially if several tightly linked SNPs within one gene are analyzed as in the present study, we also present the uncorrected p-values and discuss them as hypothesis-generating.

## Results

**Figure 1:**
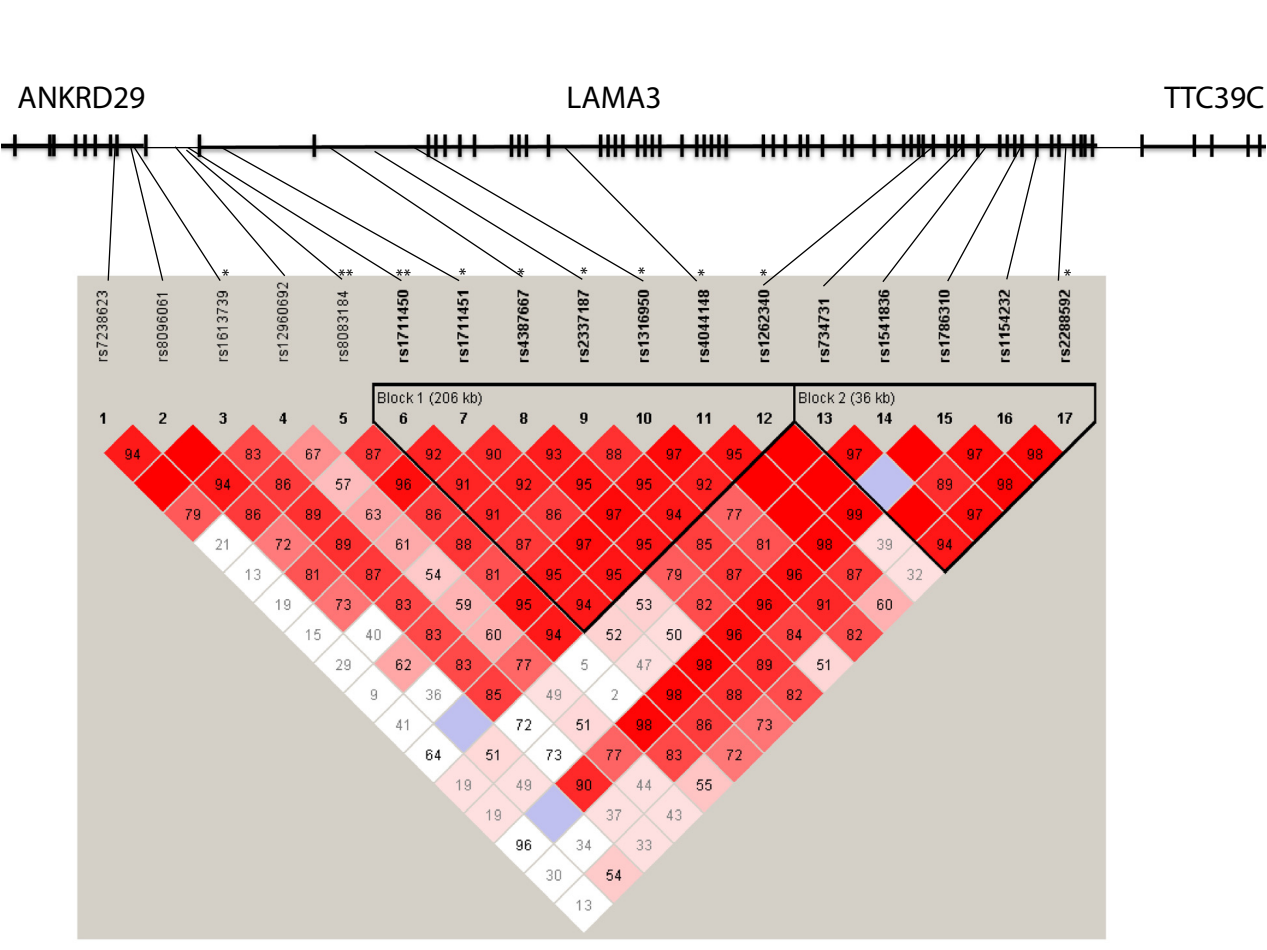
**Haploblock structure of the*LAMA3*region as revealed by Haploview 4.0 **[19]**.** *SNPs associated with AD at p < 0.01 (uncorrected values) **SNPs associated with AD at p < 0.001 (uncorrected values).

All 29 SNPs showed genotypic distributions according to HWE. Of the 17 SNPs chosen for the *LAMA3* gene, ten showed significant associations (p < 0.01 in the uncorrected analysis) with AD in the present cohort. The most significant results that also survived Bonferroni correction were obtained for rs8083184 and rs1711450, both located in the promoter region of *LAMA3* (p = 0.0003, p_corr_ = 0.0087, Table 1; full genotype data is presented in Additional file 2). A high degree of linkage disequilibrium (LD) was observed for the SNPs in the *LAMA3* gene region (Figure 1). Significant association extended into the 5′ region of *LAMA3*, including rs1613739 which is located in the neighbouring *ANKRD29* gene. Two other SNPs in *ANKRD29* (rs7238623 and rs8096061), however, did not show significant association results. Haplotype analyses revealed the existence of two haploblocks (Figure 1) with a common haplotype in block 1 that was highly significantly associated with AD (61.9% in cases *vs*. 51.1% in controls, p = 2.94×10^-5^, Table 2).

**Table 1:** **Allele frequencies of *LAMA3, LAMB3 *** **and *LAMC2 *** **polymorphisms in AD patients and controls**

**Gene**	**SNP**	**Location**	**Amino acid exchange**	**MAF in AD patients**	**MAF in controls**	**Uncorrected p-value**	Corrected p-value*
*LAMA3* 18q11.2	rs7238623	5′UTR (*ANKRD29*)	-	0.107	0.083	0.123	n.s.
	rs8096061	5′UTR (*ANKRD29*)	-	0.036	0.044	0.441	n.s.
	rs1613739	5′UTR (*ANKRD29*)	-	0.143	0.200	**0.003**	0.087
	rs12960692	5′UTR	-	0.439	0.454	0.568	n.s.
	rs8083184	5′UTR	-	0.297	0.386	**0.0003**	**0.0087**
	rs1711450	5′UTR	-	0.319	0.410	**0.0003**	**0.0087**
	rs1711451	Intron 1	-	0.336	0.408	**0.005**	0.145
	rs4387667	Intron 2	-	0.336	0.408	**0.005**	0.145
	rs2337187	Intron 2	-	0.250	0.325	**0.001**	**0.029**
	rs1316950	Intron 2	-	0.331	0.407	**0.003**	0.087
	rs4044148	Intron 12	-	0.234	0.395	**0.007**	0.203
	rs1262340	Intron 44	-	0.143	0.196	**0.006**	0.174
	rs734731	Intron 55	-	0.079	0.104	0.096	n.s.
	rs1541836	Intron 59	-	0.086	0.106	0.195	n.s.
	rs1786310	Intron 62	-	0.076	0.103	**0.047**	**n.s.**
	rs1154232	Exon 65	Asn2815Lys	0.187	0.225	0.069	n.s.
	rs2288592	Intron 69	-	0.278	0.345	**0.005**	0.145
*LAMB3* 1q32.2	rs2566	3′UTR	-	0.283	0.271	0.611	n.s.
	rs2009292	Intron 18	-	0.323	0.336	0.617	n.s.
	rs3179860	Exon 18	Leu891Leu	0.161	0.144	0.375	n.s.
	rs12748250	Exon 17	Met852Leu	0.167	0.150	0.361	n.s.
	rs2072938	Intron 11	-	0.172	0.174	0.901	n.s.
	rs4844863	Intron 8	-	0.157	0.160	0.889	n.s.
	rs2236891	Intron 8	-	0.129	0.101	0.094	n.s.
	rs2236892	Intron 8	-	0.165	0.178	0.517	n.s.
*LAMC2* 1q25.3	rs483783	Intron 1	-	0.490	0.467	0.362	n.s.
	rs601508	Intron 1	-	0.476	0.440	0.158	n.s.
	rs2274980	Exon 3	Ser99Ser	0.166	0.173	0.723	n.s.
	rs11586699	Exon 3	Thr124Met	0.077	0.062	0.263	n.s.

*after Bonferroni correction for 29 SNPs; n.s.: not significant; bold: significant results.

**Table 2:** **Frequencies and p-values of *LAMA3 *** **haplotypes in AD patients and controls**

**Haplotype**	**Frequency in AD patients (n = 470)**	**Frequency in controls (n = 320)**	**p-value**
Block 1			
1112122	0.619	0.511	**2.9x10**^**-5**^
2221211	0.134	0.182	**0.0114**
2221212	0.086	0.088	0.8954
2222222	0.060	0.070	0.4447
1212122	0.023	0.019	0.607
1122222	0.022	0.016	0.4152
2221222	0.016	0.021	0.4906
2112122	0.012	0.025	0.0465
Block 2			
12121	0.718	0.650	**0.0043**
12112	0.113	0.110	0.8506
12212	0.074	0.106	**0.0305**
21122	0.079	0.093	0.3379
12122	0.007	0.022	**0.0083**

Bold: significant results.

In the *LAMB3* gene, neither single SNP nor haplotype analyses revealed significant association with AD in the present cohort (Table 1; haplotype data not shown). In *LAMC2,* no single SNP showed association with AD; however, haplotype analyses revealed the existence of a rare protective haplotype (4% in cases *vs*. 6.9% in controls, p = 0.01; Table 3).

**Table 3:** Frequencies and p-values of *LAMC2* haplotypes in AD patients and controls

**Haplotype**	**Frequency in AD patients (n = 470)**	**Frequency in controls (n = 320)**	**p-value**
1221	0.356	0.341	0.5252
2121	0.347	0.343	0.8557
2111	0.099	0.086	0.3998
1222	0.068	0.053	0.2136
1121	0.040	0.069	**0.0102**
1111	0.037	0.058	0.0493
2211	0.022	0.016	0.4027
2221	0.015	0.014	0.8668

Bold: significant results.

## Discussion

To our knowledge, this is the first report of an association of AD with variation in the *LAMA3* gene, encoding the alpha-chain of laminin 5. In a well-characterized German case–control cohort, we found significant association of both allelic and haplotypic frequencies in this gene with AD, suggesting that it may constitute a novel susceptibility gene for this frequent skin disease.

Of the 19 SNPs evaluated across the *LAMA3* gene, ten showed genotypic or allelic association with AD at p < 0.01 (uncorrected values). Due to the extensive LD evident at the *LAMA3* locus we were not able to further differentiate the specific disease causing variation(s) in this region. Significant association extended into the 5′ neighbouring gene *ANKRD29,* but additional SNPs in this gene did not show association with AD. The biological function of *ANKRD29,* encoding the ankyrin repeat domain-containing protein 29*,* is not yet known; however, for another protein of the same family, ANKRD17, a role in anti-bacterial innate immune pathways has been suggested [21]. For the gene located 3′ of *LAMA3* (*TTC39C*), encoding the tetratricopeptide repeat protein 39C, the biological function is not yet clear either, and since the most significant results for *LAMA3* were at the 5′ end of this large gene, we did not evaluate additional SNPs in the *TTC39C* gene. Taken together, even though we cannot exclude that the observed association with AD may be due to LD with a susceptibility variant in another gene in this region, our data highly points to *LAMA3* as susceptibility gene for AD in the 18q11.2 region. SNPs in the genes encoding the beta- and gamma-chains of laminin 5, on the other hand, did not show convincing evidence for association with AD in this analysis. However, haplotype analysis suggested the existence of a rare protective haplotype in *LAMC2*.

The main role of laminin 5 in normal tissues is the maintenance of epithelial-mesenchymal cohesion in tissues exposed to external forces, such as skin and stratified squamous mucosa [22]. Genetic variation in laminin 5 components may thus contribute to reduced skin integrity and barrier function, as has been observed for *FLG* mutations [5], even though the exact underlying mechanisms still have to be elucidated. In each of the three genes analysed here, rare null mutations are known to cause epidermolysis bullosa junctionalis, a severe autosomal recessive skin disorder characterized by the development of blisters and skin erosions in response to minor injury or friction [10]. In contrast, we observed association of common variation in *LAMA3* with the common complex disorder AD. This phenomenon is in line with findings for the *SPINK5* gene, in which null mutations cause autosomal recessive Netherton syndrome while common variations have been associated with AD [23].

Our association results further contribute to the hypothesis that an intact skin barrier function plays a key part in AD pathogenesis. Additional to the established role of *FLG* mutations [24], associations with some other skin-related genes have been described recently. For example, mutations in the claudin-1 gene (*CLDN1*), encoding a major tight junction protein in the granular layer of the epidermis, were associated with AD in North American cohorts of both European and African American origin [25]. Further, polymorphisms near the *OVOL1* and *ACTL9* genes, both involved in epidermal proliferation and differentiation, showed genome-wide significant association with AD in a large meta-analysis of GWAS data, including 5,606 AD patients and 20,565 controls [26]. In a study cohort comprising AD patients from Germany, Poland and the Czech Republic, a 24-bp deletion in the gene encoding small proline-rich protein 3 (*SPRR3*), located within the EDC, was significantly associated with disease risk [27]. On the other hand, a deletion of the cornified envelope 3B and 3C genes within in the EDC was not associated with AD in a European cohort [28]. All of these results still await replication in independent cohorts. Altogether, though, evidence is accumulating that additional genes involved in epidermal differentiation and stability may be important for AD pathogenesis.

We are conscious of the fact that the cohort sizes of the present study are comparatively small so that the statistical power is only moderate. Furthermore, only for two SNPs in *LAMA3* results remained significant after strict Bonferroni correction for multiple testing, leaving open the risk for false-positive results. However, the Bonferroni correction has been controversially discussed for genetic case–control studies [20], especially if several tightly linked SNPs within one gene are analyzed, as was the case in the present study. Therefore, we regard the results as hypothesis-generating and strongly encourage replication in additional cohorts.

## Conclusions

We presented initial evidence for association of *LAMA3* variation with AD, suggesting that variation in this gene may contribute to skin fragility and impaired barrier function underlying AD pathogenesis. Additional studies in independent populations as well as functional analyses of the associated variations appear warranted to replicate or extend these findings, since the genetic risk factors for AD might be increasingly included into prognostic and therapeutic strategies in the future. In more detail, an integrated approach including genotypic and phenotypic information as well as genetic and biological biomarkers has been proposed for example to identify patients who are prone to develop persistent AD and/ or additional asthma and start early intervention [29]. Further, therapeutic strategies targeting the skin barrier function are already under way [30]. Thus, elucidating the complex genetic background of AD is essential for paving the way towards a more individualized therapy in the future.

## Abbreviations

AD: Atopic dermatitis; EDC: Epidermal differentiation complex; HWE: Hardy-Weinberg equilibrium; LD: Linkage disequilibrium; SNP: Single nucleotide polymorphism.

## Competing interests

The authors declare that they have no competing interests.

## Authors’ contributions

SS was in charge of study design and statistical analysis and drafted the manuscript. QP and EPP were involved in patient recruitment and helped to draft the manuscript. JTE and SH conceived of the study, participated in its design and coordination and helped to draft the manuscript. All authors read and approved the final manuscript.

## Pre-publication history

The pre-publication history for this paper can be accessed here:

http://www.biomedcentral.com/1471-5945/14/17/prepub

## Supplementary Material

Additional file 1 **Primers, PCR conditions and restriction enzymes used for genotyping of polymorphisms in the *LAMA3, LAMB3*** **and** ***LAMC2*** **genes.** Click here for file

Additional file 2 Complete single SNP association results. Click here for file
