# Supplementary material for: Association of variation in the LAMA3 gene, encoding the alpha-chain of laminin 5, with atopic dermatitis in a German case–control cohort
Source: BMC Dermatol. 2014 Nov 3;14:17. doi: 10.1186/1471-5945-14-17 (PMC4221780; doi:10.1186/1471-5945-14-17)
Supplement: Additional file 1 — Primers, PCR conditions and restriction enzymes used for genotyping of polymorphisms in the LAMA3, LAMB3 and LAMC2 genes. [file 1471-5945-14-17-S1.docx]

Additional file 1. Primers, PCR conditions and restriction enzymes used for genotyping of polymorphisms in the *LAMA3, LAMB3* and *LAMC2* genes

| Gene | Polymorphism | Primer sequences | Restriction enzyme | Annealing temp. (°C) |
| --- | --- | --- | --- | --- |
| *LAMA3* | rs7238623 | F: CTGAGGTTGGAATTTTCGATGTA  R: CATTTTGAAGATGAAGACTTTTAAACA | Nlalll | 53 |
|  | rs8096061 | F: CTAGAGGTCAGGAATCTTGAGC  R: TCAAGCAAGAGACTGCCTGTTG | Tru1I | 56 |
|  | rs16113739 | F: ACCCTTTCAATCGAGGCAAATC  R: GGACTGTCGCCGGGTAACAG | Eco0109I | 56 |
|  | rs12960692 | F: GCTTAGATTTGCAGGGACCTCAG  R: TTTGAGAGGTTTGTGGCTCCTC | SatI | 56 |
|  | rs8083184 | F: CTGATCCCAGTTCATATTCCTAGG  R: ATGGGTAATAAACTCAGTGTCTTACCA | Ncol | 58 |
|  | rs1711450 | F: CAGCATTGCTAATCCAAAGTCATG  R: CACTGGATTTTCAACAGGGTGC | HpyCH4V | 65 |
|  | rs1711451 | F: GCCTTATTCGGAGAAGTCGGT  R: TGAGGCTCGGATCTCTGAGAG | Hintl | 56 |
|  | rs4387667 | F: GAGAGCCTTCATGGTTCAAAAGA  R: GCACCAGAATCACTTGAGCGT | Taql | 56 |
|  | rs2337187 | F: TGAACCAGTGCTTCCCAAACC  R: CATCCTACTACTAGCCCCAGCCT | Hphl | 58 |
|  | rs1316950 | F: GCAGAAGGAGTGATTCAACAAGC  R: CCTGGAATCTTGCCAAGGTCT | Nlalll | 65 |
|  | rs4044148 | F: ATTACTGCAGGTGAAGAGGAAGGC  R: GTCATGCTCTTGAATACCTTCCAGG | Eco57l | 58 |
|  | rs1262340 | F: GAGACCCAGGCCAAGGACCT  R: GGCTCGATGAGAAAAACTCACCT | Mboll | 58 |
|  | rs734731 | F: TGAGCCTCAGAATCTGCCCTT  R: GTCTGGCTTGGTTAAACAGCTGT | HpyCH4V | 56 |
|  | rs1786310 | F: ACAGCAGGAAGTGGGATAGGGA  R: CAGCCACTCTACCTGCCTCTG | Bsp 143I | 56 |
|  | rs1154232 | F: CATTTGGGTCAAAACATCCTGAT  R: CTCACCCAGAGTTGTCGCTTATT | BspI | 56 |
|  | rs2288592 | F: CCGGAGGAAAAAATCTCTCATCT  R: GGTAAACTGGCGCTCTGATGC | BsaAI | 56 |
| *LAMB3* | rs2566 | F: CACCTGCAAGTGATGCTACAGC  R: AAGTGTAACTGTCCCATTGGCTC | Satl | 58 |
|  | rs3179860 | F: GCCTCGGCTGCACTTACGA  R: TTGGGTAGCCTCTTTAACACCG | Mbol | 56 |
|  | rs4844863 | F: TGTTGATTACTCCTGATGGCGGA  R: GCACTCGACCCAGACCCTACA | FokI | 58 |
|  | rs2072938 | F: CAGGTGGCTGGAACTGGTGG  R: CACTCTCCTCGTCACACGGC | Hphl | 56 |
|  | rs2009292 | F: ATTAGGGCAGCCGAGGAATCT  R: GCTGGCTGATGCACTGAACAT | Taql | 58 |
| *LAMC2* | rs483783 | F: CGATGGTCTCAGGTCTCCTGA  R: CAAATGGAGACTTCTGAAAAGACG | DraI | 56 |
|  | rs601508 | F: AATGAGCTGCCCTTATGAAGTTAATT  R: CAACATAGTGAAACCCCGTCTCTA | TaaI | 56 |
|  | rs2274980 | F: CCTTGTGGGTTTCAGGTTCTCT  R: AGCAGTGTTTGGCACAGGGTAG | MspI | 56 |
|  | rs11586699 | F: CCTTGTGGGTTTCAGGTTCTCT  R: AGCAGTGTTTGGCACAGGGTAG | MsiI | 56 |
|  |  |  |  |  |
