# Supplementary material for: Association of variation in the LAMA3 gene, encoding the alpha-chain of laminin 5, with atopic dermatitis in a German case–control cohort
Source: BMC Dermatol. 2014 Nov 3;14:17. doi: 10.1186/1471-5945-14-17 (PMC4221780; doi:10.1186/1471-5945-14-17)
Supplement: Additional file 2 — Complete single SNP association results. [file 1471-5945-14-17-S2.doc]

Additional file 2: Complete single SNP association results.

| **Gene** | **SNP** | **Genotype** | **Allele** | **AD patients** | **Controls** | **p-value^a^** |
| --- | --- | --- | --- | --- | --- | --- |
| *LAMA3 (ANKRD29)* | rs7238623 | GG  GA  AA  n | G  A  n | 2 (0.4%)  96 (20.5%)  370 (79.1%)  **468**  100 (10.7%)  836 (89.3%)  **936** | 1 (0.3%)  51 (16.0%)  266 (83.6%)  **318**  53 (8.3%)  583 (91.7%)  **636** | 0.112  0.123 |
| *LAMA3 (ANKRD29)* | rs8096061 | CC  CA  AA  n | C  A  n | 435 (92.9%)  32 (6.8%)  1 (0.2%)  **468**  902 (96.4%)  34 (3.6%)  **936** | 290 (91.2%)  28 (8.8%)  0 (0.0%)  **318**  608 (95.6%)  28 (4.4%)  **636** | 0.312  0.441 |
| *LAMA3 (ANKRD29)* | rs1613739 | GG  GC  CC  n | G  C  n | 334 (73.4%)  112 (24.6%)  9 (2.0%)  **455**  780 (85.7%)  130 (14.3%)  **910** | 197 (62.5%)  110 (34.9%)  8 (2.5%)  **315**  504 (80.0%)  126 (20.0%)  **630** | **0.006***  **0.003*** |
| *LAMA3* | rs12960692 | TT  TC  CC  n | T  C  n | 133 (29.3%)  243 (53.5%)  78 (17.2%)  **454**  509 (56.1%)  399 (43.9%)  **908** | 86 (27.2%)  173 (54.7%)  57 (18.0%)  **316**  345 (54.6%)  287 (45.4%)  **632** | 0.813  0.568 |
| *LAMA3* | rs8083184 | GG  AG  AA  n | G  A  n | 41 (8.8%)  196 (41.9%)  231 (49.4%)  **468**  278 (29.7%)  658 (70.3%)  **936** | 47 (15.6%)  139 (46.0%)  116 (38.4%)  **302**  233 (38.6%)  371 (61.4%)  **604** | **0.001***  **0.0003*** |
| *LAMA3* | rs1711450 | TT  CT  CC  n | T  C  n | 217 (46.4%)  203 (43.4%)  48 (10.3%)  **468**  637 (68.1%)  299 (31.9%)  **936** | 102 (34.5%)  145 (49.0%)  49 (16.6%)  **296**  349 (59.0%)  243 (41.0%)  **592** | **0.001***  **0.0003*** |
| *LAMA3* | rs1711451 | AA  AC  CC  n | A  C  n | 201 (43.7%)  209 (45.4%)  50 (10.9%)  **460**  611 (66.4%)  309 (33.6%)  **920** | 99 (33.8%)  149 (50.9%)  45 (15.4%)  **293**  347 (59.2%)  239 (40.8%)  **586** | **0.02***  **0.005*** |

| **Gene** | **SNP** | **Genotype** | **Allele** | **AD patients** | **Controls** | **p-value** |
| --- | --- | --- | --- | --- | --- | --- |
| *LAMA3* | rs4387667 | AA  AC  CC  n | A  C  n | 205 (43.9%)  210 (45.0%)  52 (11.1%)  **467**  620 (66.4%)  314 (33.6%)  **934** | 106 (33.6%)  156 (51.3%)  46 (15.1%)  **304**  360 (59.2%)  248 (40.8%)  **608** | **0.01***  **0.005*** |
| *LAMA3* | rs2337187 | AA  AG  GG  n | A  G  n | 30 (6.5%)  171 (37.0%)  261 (56.5%)  **462**  231 (25.0%)  693 (75.0%)  **924** | 36 (12.1%)  126 (40.7%)  140 (47.1%)  **297**  193 (32.5%)  401 (67.5%)  **594** | **0.006***  **0.001*** |
| *LAMA3* | rs1316950 | CC  CT  TT  n | C  T  n | 207 (44.2%)  212 (45.3.0%)  49 (10.5%)  **468**  626 (66.9%)  310 (33.1%)  **936** | 103 (34.2%)  151 (50.2%)  47 (15.6%)  **301**  357 (59.3%)  245 (40.7%)  **602** | **0.009***  **0.003*** |
| *LAMA3* | rs4044148 | CC  CT  TT  n | C  T  n | 26 (5.7%)  163 (35.4%)  271 (58.9%)  **460**  215 (23.4%)  705 (76.6%)  **920** | 24 (7.9%)  131 (43.2%)  148 (48.8%)  **303**  179 (39.5%)  427 (70.5%)  **606** | **0.02***  **0.007*** |
| *LAMA3* | rs1262340 | AA  AG  GG  n | A  G  n | 10 (2.1%)  114 (24.4%)  343 (73.4%)  **467**  134 (14.3%)  800 (85.7%)  **934** | 9 (3.1%)  98 (33.2%)  188 (63.7%)  **295**  116 (19.6%)  474 (80.3%)  **590** | **0.02***  **0.006*** |
| *LAMA3* | rs734731 | GG  AG  AA  n | G  A  n | 398 (85.2%)  64 (13.7%)  5 (1.1%)  **467**  860 (92.1%)  74 (7.9%)  **934** | 243 (80.2%)  57 (18.8%)  3 (1.0%)  **303**  543 (89.6%)  63 (10.4%)  **606** | 0.057  0.096 |
| *LAMA3* | rs1541836 | TT  TC  CC  n | T  C  n | 6 (1.3%)  67 (14.6%)  387 (84.1%)  **460**  79 (8.6%)  841 (91.4%)  **920** | 2 (0.7%)  59 (19.8%)  237 (79.5%)  **298**  63 (10.6%)  533 (89.4%)  **596** | 0.063  0.195 |
| *LAMA3* | rs1786310 | CC  CT  TT  n | C  T  n | 395 (85.3%)  66 (14.3%)  2 (0.4%)  **463**  856 (92.4%)  70 (7.6%)  **926** | 240 (80.0%)  57 (19.0%)  3 (1.0%)  **300**  558 (89.7%)  64 (10.3%)  **622** | 0.075  0.047 |
| *LAMA3* | rs1154232 | AA  AC  CC  n | A  C  n | 19 (4.1%)  135 (29.2%)  309 (66.7%)  **463**  173 (18.7%)  753 (81.3%)  **926** | 18 (5.9%)  102 (33.2%)  187 (60.9%)  **307**  138 (22.5%)  476 (77.5%)  **614** | **0.207**  **0.069** |
| *LAMA3* | rs2288592 | CC  CT  TT  n | C  T  n | 247 (53.6%)  172 (37.3%)  42 (9.1%)  **461**  666 (72.2%)  256 (27.8%)  **922** | 131 (42.8%)  139 (45.4%)  36 (11.8%)  **306**  401 (65.5%)  211 (34.5%)  **612** | **0.014***  **0.005*** |
| *LAMB3* | rs2566 | TT  CT  CC  n | T  C  n | 38 (8.3%)  184 (40.1%)  237 (51.6%)  459  260 (28.3%)  658 (71.7%)  918 | 23 (7.3%)  125 (39.7%)  167 (53.0%)  315  171 (27.1%)  459 (72.9%)  630 | 0.860  0.611 |
| *LAMB3* | rs2009292 | TT  CT  CC  n | T  C  n | 51 (11.0%)  198 (42.7%)  215 (46.3%)  **464**  300 (32.3%)  628 (67.7%)  **928** | 31 (10.1%)  144 (46.9%)  132 (43.0%)  **307**  206 (33.6%)  408 (66.4%)  **614** | 0.511  0.617 |
| *LAMB3* | rs3179860 | AA  AG  GG  n | A  G  n | 326 (70.7%)  122 (26.5%)  13 (2.8%)  **461**  774 (83.9%)  148 (16.1%)  **922** | 231 (73.1%)  79 (25.0%)  6 (1.9%)  **316**  541 (85.6%)  91 (14.4%)  **632** | 0.752  0.375 |
| *LAMB3* | rs12748250 | AA  AT  TT  n | A  T  n | 13 (2.8%)  129 (27.9%)  321 (69.3%)  **463**  155 (16.7%)  771 (83.3%)  **926** | 7 (2.3%)  79 (25.5%)  224 (72.3%)  **310**  93 (15.0%)  527 (85.0%)  **620** | 0.704  0.361 |
| *LAMB3* | rs4844863 | CC  TC  TT  n | C  T  n | 12 (2.6%)  123 (26.3%)  333 (71.2%)  **468**  147 (15.7%)  789 (84.3%)  **936** | 7 (2.3%)  85 (27.4%)  218 (70.3%)  **310**  99 (16.0%)  521 (84.0%)  **620** | 0.934  0.889 |
| *LAMB3* | rs2072938 | AA  GA  GG  n | A  G  n | 16 (3.5%)  126 (27.4%)  318 (69.1%)  **460**  158 (17.2%)  762 (82.8%)  **920** | 13 (4.2%)  82 (26.5%)  215 (69.4%)  **310**  108 (17.4%)  512 (82.6%)  **620** | 0.845  0.901 |
| *LAMB3* | rs2236891 | TT  GT  GG  n | T  G  n | 353 (75.4%)  109 (23.3%)  6 (1.3%)  **468**  815 (87.1%)  121 (12.9%)  **936** | 252 (81.0%)  55 (17.7%)  4 (1.3%)  **311**  559 (89.9%)  63 (10.1%)  **622** | 0.060  0.094 |
| *LAMB3* | rs2236892 | TT  AT  AA  n | T  A  n | 12 (2.6%)  130 (27.8%)  325 (69.6%)  **467**  154 (16.5%)  780 (83.5%)  **934** | 12 (3.9%)  85 (27.7%)  210 (68.4%)  **307**  109 (17.8%)  505 (82.2%)  **614** | 0.574  0.517 |
| *LAMC2* | rs483783 | TT  TG  GG  n | T  G  n | 121 (25.9%)  235 (50.2%)  112 (23.9%)  **468**  477 (51%)  459 (49%)  **936** | 92 (28.9%)  155 (48.7%)  71 (22.3%)  **318**  339 (53.3%)  297 (46.7%)  **636** | 0.621  0.362 |
| *LAMC2* | rs601508 | GG  GC  CC  n | G  C  n | 131 (28.1%)  227 (48.6%)  109 (23.3%)  **467**  489 (52.4%)  445 (47.6%)  **934** | 99 (31.1%)  158 (49.7%)  61 (19.2%)  **318**  356 (56%)  280 (44%)  **636** | 0.338  0.158 |
| *LAMC2* | rs2274980 | TT  TC  CC  n | T  C  n | 14 (3.0%)  126 (27.1%)  325 (69.9%)  **465**  154 (16.6%)  776 (83.4%)  **930** | 8 (2.5%)  94 (29.5%)  217 (68.0%)  **319**  110 (17.29%)  528 (82.8%)  **638** | 0.724  0.723 |
| *LAMC2* | rs11586699 | CC  CT  TT  n | C  T  n | 399 (85.3%)  66 (14.1%)  3 (0.6%)  **468**  864 (92.3%)  72 (7.7%)  **936** | 275 (87.6%)  39 (12.4%)  0 (0%)  **314**  589 (93.8%)  39 (6.2%)  **628** | 0.474  0.263 |

^a^uncorrected p-values are given here; for Bonferroni correction, see Table 1.
